# The role of policy on fortification in food processing and value addition in Malawi and Mozambique—a systematic review

**DOI:** 10.3389/fnut.2026.1765596

**Published:** 2026-02-13

**Authors:** Lydia Jade Makonda, Orlando Nipassa, Elsa Maria Salvador

**Affiliations:** 1 Department of Chemical Engineering, Faculty of Engineering, Eduardo Mondlane University, Maputo, Mozambique; 2 Centre of Excellence in Agri-food Systems and Nutrition, Eduardo Mondlane University, Maputo, Mozambique; 3 Department of Sociology, Faculty of Arts and Social Sciences, Eduardo Mondlane University, Maputo, Mozambique; 4 Department of Biological Sciences, Faculty of Science, Eduardo Mondlane University, Maputo, Mozambique

**Keywords:** food fortification, Malawi, Mozambique, policies, strategies

## Abstract

Food fortification is a public health strategy for tackling micronutrient deficiencies in sub-Saharan Africa. This systematic review explores the policies governing food fortification in Malawi and Mozambique. By comparing policy implementation, regulatory mechanisms and outcomes, this review aims to identify best practices, challenges, and opportunities to strengthen fortification programs across the region. All applicable evidence collated from reports and articles published between 2000 and 2025, databases from key organizations, and reports/literature received from key informants were systematically reviewed. A total of 29 reports were selected based on the inclusion and exclusion criteria and subjected to risk of bias assessment. The key issues include high fortification costs, lack of technical knowledge and expertise, equipment limitations, quality control issues and regulatory compliance. Malawi’s mandatory fortification, enacted in 2011, has demonstrated higher compliance rates, supported by robust monitoring mechanisms, albeit with limited reach into informal food processing sectors that serve rural populations. Mozambique’s voluntary fortification guidelines, which became mandatory in 2016, exhibit lower compliance but greater flexibility in accommodating small and medium-scale processors. This review recommends monitoring quality and reporting, results-based implementation, stakeholder and community engagement, support and incentives to the food industries, and reduction of bureaucratic burdens to achieve effective fortification programs.

**Systematic review registration:**

https://www.crd.york.ac.uk/PROSPERO/view/CRD420251113300, identifier PROSPERO (CRD420251113300).

## 1 Introduction

Micronutrient deficiencies, often referred to as “hidden hunger,” affect over 2 billion people globally, with sub-Saharan Africa bearing a disproportionately higher burden (1). From 2025 to 2030, the global number of undernourished is expected to decrease, but 512 million people are still projected to be facing hunger in 2030, of whom nearly 60% will be in Africa (2). Food fortification, defined by the World Health Organization (WHO) as the deliberate addition of one or more micronutrients to food to improve its nutritional quality and provide public health benefits with minimal health risks, has emerged as a cost-effective strategy to combat nutrient deficiencies, particularly iron, zinc, vitamin A, and B vitamins. Large-scale Food Fortification (LSFF) increased serum micronutrient concentrations in several populations and demonstrated a positive impact on functional outcomes, including a 34% reduction in anemia, a 74% reduction in the odds of goiter a 41% reduction in the odds of neural tube defects, additionally, LSFF with vitamin A could protect nearly 3 million children per year from vitamin A deficiency (3). Globally, fortification of staples such as salt with iodine, flour with iron and folic acid, and oils with vitamin A has prevented millions of cases of neural tube defects, goiter, and blindness, contributing to a decline in hidden hunger by enhancing micronutrient intake without requiring changes in dietary habits (4). The success of fortification programs depends heavily on robust policy frameworks that mandate, regulate, and monitor the addition of essential nutrients to commonly consumed foods, including considerations for safety, technological feasibility, and consumer acceptance (5). Despite the many efforts to end malnutrition through fortification, there remains a long way to go to fully realize the benefits of fortification that translate to better nutrition outcomes. Challenges such as uneven compliance, limited coverage in rural areas, and integration with other interventions like dietary diversification persist, underscoring the need for tailored, evidence-based approaches (6).

In Malawi, the magnitude of micronutrient deficiencies remains significant, particularly among vulnerable groups. In 2024, over 2.85 million children under the age of five were screened for child wasting and nutrition oedema across all 19 El Niño-affected districts (7). Approximately 37% of children under 5 years are stunted, indicating chronic malnutrition, with wasting at 2.7% (8). Anemia statistics indicate 63% of children aged 6–59 months and 33% of women aged 15–49 are anemic, whereas one-third of women with a child born in the past 5 years took iron tablets for 90 days or more during the pregnancy of their last child to boost iron levels (8). Disaggregation by gender shows anemia rates are higher in women than men, and boys often face elevated risks of stunting compared to girls, influenced by cultural and socioeconomic factors (9, 10). These deficiencies affect an estimated 1.5 million children under 5 for stunting and anemia, exacerbating issues amid climate vulnerabilities. To address this, Malawi has implemented mandatory fortification programs since the early 2000s, including iodization of salt, fortification of sugar and cooking oil with vitamin A, and maize and wheat flour with iron, zinc, and B vitamins, supported by national policies and partnerships like Project Healthy Children (11). The programs included the Salt Iodization Act of 1999, The Malawi Gazette Supplement, dated 27th March, 2014 (12). These set out the standards, rules and regulations for the production of fortified food vehicles namely salt, sugar, wheat flour, maize flour and cooking oil. Research suggests these efforts could meet micronutrient requirements for the at-risk populations if compliance is improved; however, challenges like monitoring and rural access limit full impact.

In Mozambique, malnutrition burdens are similarly acute; 37% of children under 5 years of age suffer from chronic malnutrition, 4% from acute malnutrition, 15% are underweight, and 3% are overweight (13). The DHS survey also indicated 73% of children aged 6 to 59 months and 52% of women aged 15–49 have anemia (13). Iron deficiency affects 38.9% of children and 25.1% of women, with iron-deficiency anemia at 27.8% in children and 16.1% in women (14). Zinc deficiency stands at 17.3% in children and 13.5% in women (10). Overall, these issues impact over 2 million children under 5 and 54% of households are unable to afford nutritious diets (15). Mozambique’s fortification efforts were formalized through a 2016 landmark law (Decreto n. 9/2016 de 18 de Abril) with the objective of the regulation being to establish the regime applicable to the mandatory addition of micronutrients to the food products. It mandates the addition of micronutrients to wheat and maize flour (iron, zinc, folic acid, B vitamins), sugar (vitamin A), and edible oils (vitamin A and D).

Existing policies often face challenges like poor compliance, inadequate monitoring, and limited integration with dietary diversification efforts, resulting in no comprehensive repository of good practices for scaling up fortification in sub-Saharan Africa (6). This work is timely and desirable amid rising malnutrition trends exacerbated by climate events, economic pressures, and the approaching 2030 SDG deadline, where persistent deficiencies underscore the need for evidence-based policy refinements to enhance nutritional security and economic growth (16). The primary objective of this work is to conduct a comprehensive comparative analysis of food fortification policies in Malawi and Mozambique, examining their development, implementation, and impact on food processing and value addition sectors and in doing so identify best practices, challenges and opportunities.

### 1.1 Research question

What are the key differences and similarities in food fortification policies between Malawi and Mozambique, and how have these policies influenced food processing and value addition in each country?

The research hypotheses are (1) Countries with more comprehensive and well-enforced fortification policies will demonstrate greater compliance rates among food processors and better public health outcomes related to micro-nutrient deficiencies; and (2) There is no significant relationship between the comprehensiveness of fortification policies and compliance rates or public health outcomes.

This manuscript begins with an introduction outlining the background and objectives of the study, followed by a detailed description of the materials and methods used. The subsequent section presents the results, which are then discussed in relation to existing literature and the study’s aims. Finally, the manuscript concludes with key findings and their implications, alongside policy recommendations.

## 2 Materials and methods

### 2.1 Scope of the review

The scope of this systematic review covers the geographic, sectoral, thematic and food categories.

Geographical scope: This systematic review focuses specifically on food fortification policies in Malawi and Mozambique, examining national-level policies, regulations, and implementation frameworks from 2000 to 2025.

Sectoral scope: The review encompasses policies affecting large-scale commercial food processing industries, small and medium-scale food processors, informal food processing sectors, import and export regulations for fortified foods, quality control and monitoring systems, and public-private partnerships in fortification.

Thematic scope: key thematic areas include mandatory versus voluntary fortification policies, regulatory frameworks and enforcement mechanisms, standards and specifications for fortified foods, monitoring and evaluation systems, economic incentives and disincentives including subsidies, capacity-building and technical assistance programs, consumer awareness and education policies, and trade and import/export regulations.

Food categories: The review examines fortification policies for wheat flour, maize flour, vegetable oils, sugar, and salt.

This analytic framework of this systematic review was guided by the population, intervention, comparators, outcome and setting (PICOTS) framework (Table 1).

**Table 1:** PICOTS framework.

Component	Description	Design for comparative study (Malawi vs. Mozambique)
Population / problem	Food processors and value chain stakeholders that are involved in the production of staple fortified foods (maize flour, wheat flour, salt, sugar, oil). The problem is the limited or varying effectiveness of national policies on food fortification.	Food processing companies, policy makers, health agencies, and smallholder processors in Malawi and Mozambique.
Intervention	Implementation and enforcement of food fortification policies and programs (mandatory fortification, subsidies, technical support, regulation, capacity building).	Review of national policies, implementation mechanisms, public-private partnerships, and technical support in both countries.
Comparator	Comparative country context—differences and similarities in policy design, enforcement, stakeholder participation, and monitoring systems.	Malawi’s fortification framework against Mozambique’s framework (voluntary/mandatory levels, regional standards).
Outcomes	Increased compliance with food fortification standards Improved micronutrient intake and public health Enhanced value addition and market competitiveness Processor-level economic performance	Comparing the following outcomes: Fortification compliance rates Nutritional outcomes (proxy data) Economic benefits to processors Market expansion in each country

### 2.2 Search strategy

A search for studies published between 2000 and 2025 was conducted using the following keywords: “food fortification” AND “policy” AND (“Malawi” OR “Mozambique”), “food fortification” AND “strategies” AND (“Malawi” OR “Mozambique”). The websites for the search included Google Scholar, PubMed, ResearchGate, Semantic Scholar and Springer. Global reporting databases that contained relevant documents on fortification data, including the Global Fortification Data Exchange (GFDX), the WHO Vitamin and Mineral Nutrition Information System database, and DHS and MICS, were also included in the search. Studies reporting in the Portuguese and English languages were included.

### 2.3 Inclusion criteria

- Papers discussing fortification in Malawi and Mozambique, regional and global studies that included either one or both of the two countries.
- Fortification vehicles: salt, sugar, maize flour, wheat flour and cooking oil.

### 2.4 Exclusion criteria

**Figure 1:**
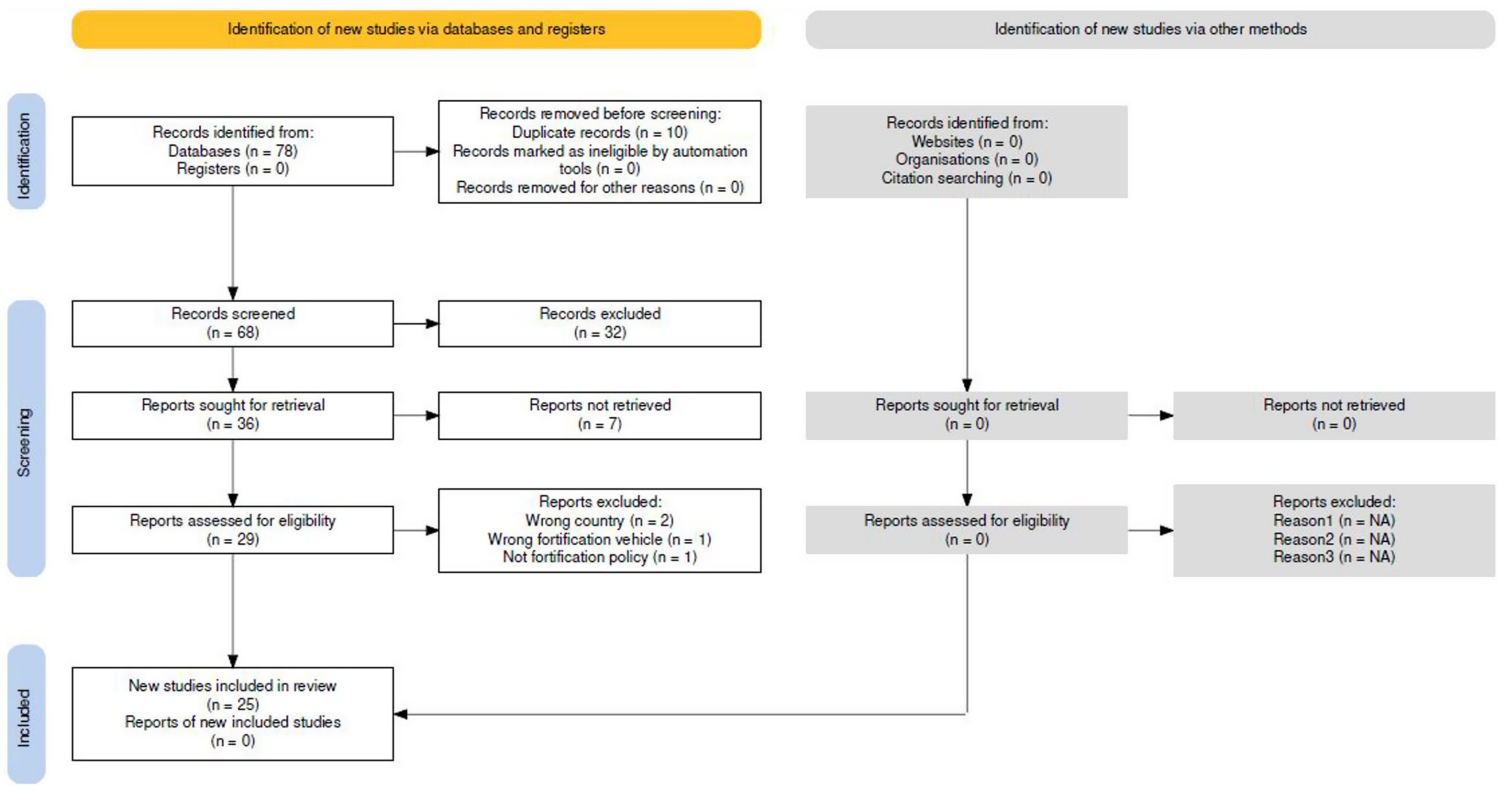
PRISMA flowchart of the literature search for selection and inclusion of articles and reports.

- Studies covering rice fortification (voluntary and not mandatory fortification).
- Biofortification programs (crop breeding approaches),
- Micronutrient supplementation programs,
- Dietary diversification interventions
- Policies from other countries in the region and implementation outcomes at sub-national levels, unless directly related to national policy frameworks (Figure 1).

### 2.5 Data extraction

Data extraction was performed independently by one reviewer using a standardized, pilot-tested Excel extraction form developed specifically for this review. The extraction form captured study characteristics, including title, author, journal name, DOI, publication year, setting, suggested solutions, key challenges, and key findings. All selected articles were run through Scispace, an AI tool designed for data extraction to check for accuracy before analysis. The articles were also entered into Mendeley and Zotero and standardized for easy tracking, citation, and referencing articles and reports during the review.

## 3 Results

Out of 78 total gathered publications, 25 studies that complied with the inclusion/exclusion criteria were included in this study and are shown in Table 2.

**Table 2:** Evidence table.

Source	Suggested solution	Key challenges	Key findings
Rowe (6)	Premix reconciliation calculation	Current monitoring systems	Effective data collection
Asirvatham et al. (55)	New nutrition-sensitive policies	Old policies	Improved policy coordination and collaboration
Mildon et al. (18)	Community-based fortification	Nationwide initiatives	Improved reach of fortification initiatives
Vasta et al. (56)	Digital tools and technologies	Non-digitized tools	Improved program decision-making and nutrition impact
Tarini et al. (57)	Fortification	International community guidance, zinc deficiency assessment, regional fortification standards, and lack of evidence	Improved zinc intake
Olson et al. (49)	Improved private-public partnerships, key support in advocacy, management, implementation, capacity building, and regulatory monitoring	Current fortification programs	Improved fortification implementation
Benson et al. (58)	Agriculture and food security policies	Strong performance on policy processes 2017/2018	Improved processes from improved implementation
Rohner et al. (59)	Collection and usage of fortification program data in decision-making and program improvement	Available coverage data from salt fortification, possibly from inclusion in MICS and DHS	Improved implementation of LSFF
Makhumula et al. (60)	Model of fortification legislation		increased nutrient uptake, easy monitoring and evaluation, corrective action, and assigns roles and responsibilities
Meerman (61)	incorporating nutrition in national policies	Food security equals nutrition security	Cross-sectoral collaboration, mainstreaming the nutrition agenda
Storhaug et al. (62)	Nutritional interventions		Addressing key evidence gaps in the evaluation of national-level policies evaluation
Bell et al. (54)	Revising guidelines on regulations	Codex/RSA	Harmonized regulation eases fortification implementation
Wessells et al. (63)	Large-scale food fortification (zinc)	Current LSFF programs	Zinc inclusion in LSFF, boosting current zinc fortification levels
Babu et al. (38)	Local leadership, coordinating power in the policy hierarchy	Process triggered by external events, external funding, no concrete strategy for biofortification, poor physical infrastructure and monitoring for LSFF	Better implementation
Tang et al. (37)	Mathematical modeling framework	Current tracking methods	LSFF is beneficial for mostly urban populations, with low consumption of some vehicles
Della Lucia et al. (64)	Correct choice of the fortifying micronutrients, Establishment of fortification levels and the selection of a suitable vehicle		Effective fortification
Mejia et al. (65)	National micronutrient fortification commissions or alliances aimed to foster inter-program coordination	No provisions in the existing regulations require coordination mechanisms among interventions	A comprehensive regulatory framework for coexisting micronutrient interventions
Reme (66)		cross-sectoral coordination, nutrition awareness, continued government support, capacity-related issues, lack of institutional home and funding	
Hess et al. (67)	FRAT	Recommended sampling reconsidered, guidelines revised to clarify important aspects of fieldworker training, implementation, data analysis and interpretation and reporting of the results.	National food fortification planning
Marks et al. (5)	Checklist for fortification policy and programs	Roles and responsibilities between agencies, the cost of regulating fortification, and enforcement strategies are often lacking.	Improved regulations and implementation
Bell et al. (47)	Dietary diversification	Compulsory fortification is excessive, unproductive, and likely harmful to human health	Eliminates the underlying causes of malnutrition, unlike mandatory fortification
Mkambula et al. (11)	New LSFF programs, Implementation research, advocacy, and new vehicles	Current LSFF programs	
Mkambula et al. (68)	Fortification and biofortification	needs, constraints, and opportunities of the population in terms of consumption patterns, supply chains, and market structures; (2) easy-to-implement, cost-effective and real-time monitoring of program delivery, coverage, cost, and nutrient intake and a rigorous evidence-based approach, including lessons learnt, to help inform policy	
Han et al. (69)		The agronomic, economic, and administrative capability of the target regions regarding fortification methods and vehicles to maximize effectiveness. Strategies to ensure the producers’ and consumers’ adoption to enhance the uptake rate, evaluate outcomes for economic metrics rather than focusing solely on before-and-after comparison to avoid biased assessment.	
Lalani et al. (70)	Business models and approach	Current approach	

Tool used	Key domains	Risk of bias judgment (Low/Moderate/High)	Notes/Justification
ROBINS-I	Confounding | Participant selection | Intervention classification | Deviations from interventions | Missing data | Measurement of outcomes | Selective reporting		
CASP (or JBI)	Clear research aim | Appropriate methodology | Rigorous data collection | Researcher bias minimized | Robust analysis | Credible findings		
AACODS	Authority | Accuracy | Coverage | Objectivity | Date | Significance		
CASP		Moderate	Researcher bias minimisation not clear
CASP		Moderate	Ethical consideration, not clear
CASP		Low	
CASP		Low	
CASP		Low	Rigorous participant selection, data collection and analysis
CASP systematic		High	Paper selection not so clear, research bias minimisation not clear
AACODS		High	No bibliography, no peer review
CASP systematic		Low	Robust data collection and analysis, risk of bias, measurement if results
CASP		Moderate	Paper selection not so clear
AACODS		High	Date not clear, no peer review
CASP systematic		Low	Researcher bias minimisation clear
CASP		Moderate	Research methodology not clear
CASP		Moderate	Research methodology not clear
AACODS		Low	
CASP		Moderate	Measurement errors
CASP		Moderate	Paper selection not clear, risk of bias minimisation not clear
CASP		Moderate	Risk of bias minimisation not clear
AACODS		Low	
CASP		Low	Rigorous participant selection, data collection and analysis
CASP		Low	
CASP		High	Aim not clear, Research methodology not clear
CASP		Low	
AACODS		Low	
CASP		Low	
CASP		Low	

### 3.1 Risk of bias assessment

The risk of bias assessment is summarized in Supplementary Table S1. Among the included studies, 13 were judged as low risk of bias, 8 had some concerns, and 4 were at high risk. The domains most frequently affected where research bias minimization was not clear, and research methodology was not clear. Whereas rigorous participant selection, data collection and analysis were generally well addressed. No clear relationship could be established between the year of publication and the setting in relation to the occurrence of risk of bias. Overall, the presence of some high and moderate risk of bias across several studies indicates that the strength of the evidence should be interpreted with caution.

### 3.2 Policy development: policy timeline

#### 3.2.1 Malawi

Malawi has been involved in food fortification efforts, but the policy evolution appears less structured compared to Mozambique. The Food Fortification Initiative (FFI) database shows data for Malawi, but specific legislative milestones and timelines are not as clearly documented in the available sources. Malawi lacks the same level of documented policy clarity in the available sources, though this may reflect documentation gaps rather than actual policy deficiencies ie a lack of implementation.

The key drivers for Malawi were mainly food security challenges in Malawi dating back to the country’s food crisis of 1991 and 1992, when a drought in southern Africa severely reduced maize production. Climate vulnerability is also prominent, as most livelihoods depend on rain-fed agriculture, making the population highly vulnerable to disasters, particularly droughts and cyclone-induced floods. Despite facing numerous challenges, Malawi has made notable progress in reducing hunger since 2000, as measured by the Global Hunger Index, which tracks overall hunger and undernutrition levels. Malawi appears to have a less formalized comprehensive framework based on available evidence, although large-scale food fortification may be a cost-effective intervention to increase micronutrient supplies in the food system when implemented under appropriate conditions. This can be supported by modeling food fortification contributions to micronutrient requirements in Malawi using Household Consumption and Expenditure Surveys (17).

#### 3.2.2 Mozambique

The2016 landmark law (Decreto n. 9/2016 de 18 de Abril) calls for fortification of five food vehicles: wheat flour, maize flour, cooking oil, sugar, and salt. Specifically, wheat flour and maize flour are to be fortified with iron, zinc, folic acid, and vitamin B12, and optionally with vitamin A, thiamine, riboflavin, niacin, and vitamin B6. Cooking oil and sugar will be fortified with vitamin A, while salt will be fortified with iodine. This encompasses the policy and regulatory scopes. The key drivers for the mandate were the high malnutrition rates that led to the development of this policy as part of a comprehensive strategy to address chronic undernutrition. International partnerships, including the Global Alliance for Improved Nutrition (GAIN), Helen Keller International (HKI), Irish Aid, the United Nations Children’s Fund (UNICEF), the Food and Agriculture Organization of the United Nations (FAO), the World Food Program, and Population Services International (PSI), also played a role in pushing the fortification agenda. Technical capacity building by Smarter Futures, where representatives from Mozambique have attended multiple training events. Lastly, the multi-sector approach ensured national leaders have included the Ministry of Industry and Commerce, the Ministry of Finance, the Ministry of Agriculture, as well as the Ministry of Health.

Mozambique’s legislation exempts the smallest producers from mandatory fortification, leaving its 13 roller maize mills, 13 hammer mills, and 10 industrial wheat mills as the main implementers of the policy. This implementation strategy follows the landmark law for mandatory food fortification. Mozambican stakeholders have been trained to use Fortification Monitoring and Surveillance (FORTIMAS) as a monitoring tool in addition to physical inspections. A clear timeline has also been set, where the law gives industries 6 months to meet the new requirements and provides detailed specifications for each food vehicle. However, exemptions are also defined where clear criteria for which producers are exempt, for example, small-scale producers are not heavily monitored and penalized. Mozambique is now seeing a structured progression from voluntary (2013) to mandatory (2016) food fortification policies.

Table 3 provides a summary of the policy timelines in Malawi and Mozambique.

**Table 3:** A summary of the policy timeline in both countries.

Malawi	Mozambique
Early 2000s: Following widespread undernutrition, the government piloted community-level fortification, supported by NGOs like World Vision	2010–2011: Post-chronic malnutrition planning led to the launch of a strategy and the creation of CONFAM, the National Committee for Food Fortification.
2008–2011: Driven by high micronutrient deficiency rates, the Department of Nutrition and HIV/AIDS initiated a national food fortification program.	2012–2015: Voluntary fortification began in 2013, with the committee laying the technical and standards groundwork, which led to the development of standards. NM 5 Maize flour, NM7 Wheat flour, NM425 Vegetable oil, NM110 Sugar and NM9 Salt
2011: Standards for mandatory fortification of maize flour, wheat flour, cooking oil, and sugar were developed. Wheat Flour MS 30 Fortified wheat flour specification (mandatory), Maize Flour MS 34 Fortified maize flour specification (mandatory) MS 202:2013 Fortified White Sugar—Specification, MS 188:2008 Edible Salt—Specification MS51:2011 Fortified Edible Oils—Specification	2016: Decree approved by the Council of Ministers mandated fortification of wheat flour, maize flour, oil, sugar, and salt, with a 180-day grace period. Mozambique published legislation on 18 April 2016 to fortify five food vehicles as part of its multi-sector plan to reduce chronic undernutrition (Decreto n. 9/2016 de 18 de Abril)
2011 onward: These standards were enshrined in the Gazette, becoming legally enforceable; inspection and monitoring systems were rolled out via the Malawi Bureau of Standards and the Ministry of Health. (The Salt Iodization Act of 1999, The Malawi Gazette Supplement, dated 27th March, 2014)	Mid-2016: Mandatory standards legally enforced; industry required to comply, backed by support from GAIN, Helen Keller International, WFP, Irish Aid, Gates Foundation, EU, and Danida.
Recent shifts: The 2018–2022 National Multi-Sector Nutrition Plan incorporated home fortification using micronutrient powders (MNPs) for infants as a complementary strategy.	Late 2016: Implementation began with inspections, equipment installations, and baseline data collection commenced.
2025: Launch of National Multisector Nutrition Policy and Strategic Plan 2025–2030	

Malawi Bureau of Standards Act, 2012, Act No 14 of 2012. Malawi. 13/February/2015.rce: Catalogue of Malawi Standards. Malawi. Instituto Nacional de Normalizicao e Qualidade (12, 71, 72).

Malawi has 2 main documents that outline their policy on fortification the Salt Iodization Act was introduced earlier in 1999, followed by the Malawi Gazette Supplement, dated 27th March, 2014 which cover sugar, maize flour and wheat flour. The Decreto n. 9/2016 de 18 de Abril from Mozambique is the law that covers the mandate to fortify all the food vehicles. Table 3 reveals that Malawi adopted mandatory food fortification earlier than Mozambique, a difference that is significant in terms of program maturity and institutional learning. Malawi’s earlier start allowed more time to integrate fortification into national nutrition strategies, establish standards, and build regulatory experience. However, the extended duration of implementation does not always result in uniformly better micronutrient outcomes due to persistent challenges in compliance, enforcement, and coverage of small-scale mills. In contrast, Mozambique’s later adoption means that population-level nutritional impacts are less evident to date, but its more centralized legal framework may enable faster gains if implementation bottlenecks are addressed. This comparison suggests that while early policy adoption can facilitate long-term capacity development, reductions in malnutrition depend on other factors like implementation quality and regulatory effectiveness than on timing alone.

### 3.3 Implementation strategies

#### 3.3.1 Malawi

Institutional coordination for the fortification process was established through the establishment of the National Fortification Alliance to bring together the government (Ministry of Health, Bureau of Standards), the private sector, NGOs, and donors. The National Fortification Alliance provides private-public oversight and coordinates compliance and feedback loops.

Government inspectors perform routine factory-level monitoring quarterly by sampling at mills and laboratories and tracking results via Malawi’s Fortification Monitoring Tool. Projects by NGOs like World Vision also champion community-level fortification piloting, where premix is added to grains in village mills or homes, relying on community structures and cost-recovery models (18).

Despite these systems being established and partially functional, compliance fluctuates due to fragmented funding and reliance on donor funding rather than stable government funding, leading to inconsistent district-level activities (11). There is also a lack of technical capacity and funding to maintain quality control standards at small mills. Small mills face limited premix supply, testing tools, and expertise hinder quality control (18). Other challenges include weak community models, home-level fortification struggled to sustain cost recovery and quality once external funding ended (18). The limited private sector engagement, leading to private sector uptake remaining low, is usually attributed to a lack of incentives (5, 19).

#### 3.3.2 Mozambique

The National Committee for Food Fortification (CONFAM) was established as part of Mozambique’s implementation strategy to coordinate multi-stakeholder governance and advocacy. This was later followed by the adoption of the FortifyMIS digital monitoring system. This mobile and online management information system allows inspectors and producers to upload data, track compliance, and generate dashboards. It was rolled out in Maputo province in 2019 for digital real-time monitoring (20). The National Inspectorate for Economic Activities (INAE) pushed the regulatory rollout through an inspectorate expansion; 30 more regulatory agents were trained in 2024 to standardize inspection protocols nationwide and improve physical oversight (21).

Effectiveness is, however, still a challenge; it was reported in 2025 by CONFAM that coverage of fortified products reached 70% of industrial production, including salt iodization at 50 to 60% market share (22). However, monitoring remains inconsistent across regions, compliance is uneven, and malnutrition rates remain high. The main challenges arise from market smuggling, unregistered maize flour from informal trade circumvents fortification (23). Evidence was suggestive that inspection capacity gaps exist due to a lack of equipment and training, small producer constraints from premix procurement and technical capacity, as well as geographic coverage and integration across sectors being weak.

Table 4 summarizes the implementation strategies used by both countries.

**Table 4:** Implementation strategy summaries of the two countries.

Aspect	Malawi	Mozambique
Governance model	National Fortification Alliance; ministry coordination	CONFAM committee with a strong private-public coalition
Monitoring system	Quarterly lab sampling; Fortification Monitoring Tool	FortifyMIS digital reporting; new inspector training
Coverage/compliance	Major mills monitored; small mills less consistent	70% industrial compliance; salt iodization 50 to 60%
Key challenges	Fragmented funding, capacity in small mills, and low private engagement	Smuggling, inspection reach, small-producer integration, data gaps

Mildon et al. (18); Mkambula et al. (11); Food Fortification Initiative (20); Club of Mozambique (21); CONFAM (22); Ministry of Industry and Commerce (23).

Overall, Table 4 demonstrates that differences in governance structure, decentralized in Malawi versus centralized in Mozambique shape how monitoring systems operate and where compliance breakdowns occur, with both systems facing shared challenges in extending regulatory reach beyond the formal industrial sector.

Malawi has functional legal and monitoring frameworks at the industrial level, supported by multi-stakeholder oversight. The Salt Iodization Act of 1999, The Malawi Gazette Supplement, dated 27th March, 2014 and Decreto n. 9/2016 de 18 de Abril provide a legal mandate, backed by the necessary standards for both countries. Yet, sustainable funding and small-mill capacity remain weak links. Mozambique excels with tech-driven monitoring and formal coordination via CONFAM and FortifyMIS, and strong industrial coverage (24). However, informal markets, inspection reach, and rural/small-producer challenges persist for both.

### 3.4 Industry response

#### 3.4.1 Malawi

Large-scale millers and processors swiftly invested in fortification equipment and sourcing premix once national standards became mandatory in 2018 (19). The Ministry of Health and the Bureau of Standards provide technical support to comply. Small and medium-scale processors (SMSPs), often cooperatives or small mills, struggle with certification and technical capacity. Rural cooperatives face bureaucratic delays (for example, year-long waits for certification) and limited ability to scale beyond local markets. Technical and financial barriers, including the high cost of premix and dosing equipment, are prohibitive for smaller processors, as they lack the economies of scale typical of large mills. The high cost and lengthy process for Bureau of Standards approval prevent expansion and broader market access (25).

In terms of influence on investment, public–private partnerships like Techno Serve’s (SAFE) initiative, supported by USAID and global food companies, have provided technical assistance and facilitated investments in fortification infrastructure for over 1,000 processors across Africa, including Malawi (26). Government grants and innovation funds have also helped processors adopt new technologies and build capacity.

#### 3.4.2 Mozambique

CONFAM coordination brought together large and medium processors, encouraging investment in dosing equipment and premix use. Digital monitoring systems (for example, FortifyMIS) incentivized investment by enabling real-time compliance tracking and making it more rewarding to invest in fortification equipment. Small miller exclusion led to mandatory regulations focusing on larger mills, while small informal hammer-mills often remain exempt or non-compliant and even though it relieves the small mills, this limits access to fortified staples in rural areas. Support mechanisms are lacking, without dosing machines or reliable premix supply, small processors cannot comply, leaving them at a disadvantage (27).

Ecosystem building through donor and NGO-backed advocacy and blended finance has attracted investments in premix blending facilities, dosing technology, and mill upgrades (27). Government industrial strategy has also created broader investments in food processing, indicating a favorable policy environment supportive of fortified food technology.

Table 5 is a summary of the industry response to the national fortification initiatives in both countries.

**Table 5:** Industry response summary for both countries.

Aspect	Malawi	Mozambique
Adoption by large firms	High uptake, supported by technical outreach	High uptake, driven by CONFAM and digital tools
SMSP impact	Production cost increases; certification barriers hamper growth	Mostly excluded; lack tech support and premix access
Technology investments	Grants and programs helping adoption; the ecosystem is growing	Investments fostered by blended finance and monitoring systems
Regulatory clarity	Legally binding, but bureaucratic processes persist	Legally clear for large mills; informal sector oversight is weak
Main challenge	Sustainability for SMSPs, bureaucratic delays	Informal sector compliance and capacity gaps

Lalani et al. (19); Kondowe (25); Technoserve, Partners in food, and USAID (26); Nelson et al. (27).

Table 5 shows that mandatory fortification has been most readily adopted by large firms in both Malawi and Mozambique, reflecting their superior technical capacity, access to finance, and closer engagement with regulatory authorities. In Malawi, adoption has been supported by technical outreach and grant-funded programs, whereas in Mozambique compliance among large firms is driven more by centralized coordination through CONFAM and digital monitoring tools. In contrast, small and medium-scale processors face substantial barriers in both countries: in Malawi, rising production costs, certification requirements, and bureaucratic delays undermine sustainability, while in Mozambique SMSPs are largely excluded due to limited technical support and restricted access to premix. These differences indicate that while fortification policies are legally clear and effective within the formal industrial sector, persistent capacity gaps and weak integration of smaller producers constrain coverage and limit the potential population-level nutritional impact.

### 3.5 Effectiveness and impact mandatory food fortification policies in Malawi and Mozambique

#### 3.5.1 Malawi

Dietary adequacy modeling before 2025 revealed that stronger enforcement of oil and sugar fortification (widely consumed staples) could significantly boost vitamin A intake, especially among higher-income and urban groups. However, less boost for poor rural populations (28). A cross-sectional study combining community-level flour fortification with dietary diversification and iron–folate supplementation reported reduced anemia over 4 years. Effectiveness could not be fully quantified due to a lack of baseline data, but it suggested a real improvement (29).

However, unintended and adverse consequences exist from equity concerns; modeling data highlight that fortified foods benefit wealthier, urban households more, leaving rural and low-income groups behind. There is also a risk of excess intake. Malawi was flagged as one of the countries at risk of vitamin A hypervitaminosis due to the overlap of supplementation and fortification (28). As well as limited iron efficacy, globally, food fortification on its own contributes modestly to iron intake, 0–13% of RNI for women, implying limited anemia impact unless programs are carefully tailored.

#### 3.5.2 Mozambique

Biofortification evidence shows that the introduction of orange-fleshed sweet potato (OFSP), a home and crop-based fortification strategy, led to a long-term increase in vitamin A intake. In one trial, women’s vitamin A intake remained higher in OFSP areas even 3 years post-intervention (29). Integrated dietary interventions, which are programs involving fortified complementary foods, IYCF counseling, and water/sanitation, showed reduced anemia risk among children in other LMIC contexts (29) Similar mixed-model approaches are implemented in Mozambique.

Bioavailability constraints from high phytate content in local diets may inhibit absorption of fortified minerals (for example, iron/zinc), reducing effectiveness unless dephytinization or complementary interventions are added. Inconsistencies in monitoring have also led to uneven fortification levels across provinces no direct evidence of nutrient toxicity yet, but the potential risk of under- or over-fortification exists (14).

Table 6 provides a summary of the effectiveness and impact of national fortification policies in both countries.

**Table 6:** Effectiveness and impact summary for both countries.

Dimension	Malawi	Mozambique
Vitamin A status	Improved via oil/sugar fortification, but the risk of hypervitaminosis exists	OFSP biofortification showed sustained Vitamin A outcome gains
Anemia reduction	Some decline noted, but anemia is multifactorial, and iron fortification impact is limited	Integrated strategies show promise, though standalone fortification data are limited
Equity	Urban/rich benefit more; rural poor lag behind	Likely similar inequity; rural access and absorption barriers unclear
Bioavailability issues	Not well-documented locally, but global evidence suggests iron absorption challenges	High-phytate diets potentially limit fortified micronutrient uptake

Bourassa et al. (28); Bechoff et al. (29); Picolo et al. (14); Keats et al. (3).

Positive gains, especially for vitamin A, anemia, and goiter (globally, LSFF reduces anemia by 34%, goiter by 74%) (3). Effectiveness hinges on context, program reach, dietary patterns, enforcement quality, and baseline nutrition status significantly influence outcomes. Risks persist from equity gaps, nutrient absorption issues, and possible overexposure; these demand careful design and continuous refinement. Integrated strategies work best by combining fortification with crop-based biofortification (for example, OFSP), supplementation, and dietary diversification, and supplementation yields more robust micronutrient improvements in children and mothers.

### 3.6 Comparative analysis

#### 3.6.1 Similarities in policy approaches

Both countries mandate fortification of key staples, including salt (with iodine), sugar (with vitamin A), cooking oil (with vitamin A), wheat flour, and maize flour (with iron, zinc, folic acid, and other micronutrients). This aligns with global best practices for reducing deficiencies in vitamin A, iodine, and iron, as seen in Sub-Saharan African programs (11). Policies prioritize industrially milled products, with standards enforced by national bodies like Malawi’s Bureau of Standards and Mozambique’s National Institute of Standards and Quality, both emphasize monitoring imports to ensure compliance (30, 31). Fortification is complemented by dietary diversification and supplementation in national nutrition policies, recognizing it as a cost-effective food-based approach to combat deficiencies.

#### 3.6.2 Differences in policy approaches

Malawi introduced mandatory fortification in 2011, covering four staples (wheat flour, maize flour, sugar, oil) with detailed standards updated by 2015 (31). Mozambique’s policy, enacted in 2016 via Decree No. 9/2016, mandates similar vehicles but includes rice in some voluntary aspects and faces delays in full implementation (32). Malawi’s earlier start ensured more mature monitoring systems. Malawi emphasizes robust regulatory enforcement at district levels and multi-stakeholder coordination, leading to higher compliance in large-scale operations. Mozambique struggles with uneven enforcement, particularly for small-scale mills, due to limited resources and bureaucratic hurdles (33). Malawi’s policies better integrate small mills through sensitization and equipment support, while Mozambique’s approach highlights gaps in covering community-level processing, leading to voluntary elements for non-mandatory vehicles like rice.

#### 3.6.3 Comparison of outcomes and effectiveness of policy strategies

Mandatory strategies in both countries have increased fortified food availability, with Malawi achieving higher household coverage for vitamin A-fortified oil and sugar. Mozambique reports 78.4% household coverage for fortified salt, but lower for maize flour due to small-scale exemptions (34). Overall, both reduce micronutrient deficiencies, but Malawi’s earlier policy correlates with better vitamin A status (35). Mandatory approaches yield higher compliance compared to voluntary, with improved public health outcomes (36). Mozambique’s partial voluntary inclusion for rice shows lower effectiveness, emphasizing the need for full mandates. Combined strategies (fortification and diversification) improve outcomes more than fortification alone. Both policies are cost-effective and beneficial in averting deficiencies; however, uneven coverage exacerbates rural–urban disparities (37).

#### 3.6.4 Analysis of factors contributing to successful policy implementation

The effectiveness of mandatory food fortification policies is determined by the extent to which regulatory requirements are implemented and enforced across the food system. While the existence of fortification mandates reflects strong political commitment, policy effectiveness depends on consistent compliance by food processors, effective regulatory oversight, and sustained coordination among public and private stakeholders.

Strong regulatory frameworks, supported by clear fortification standards, routine inspections, and enforcement mechanisms, play a critical role in ensuring compliance. Evidence indicates that countries with well-resourced regulatory agencies and systematic import monitoring achieve higher compliance rates, particularly where fortified foods are sourced from both domestic and international markets (17). Multi-stakeholder coordination—linking government ministries, standards authorities, industry associations, and development partners further enhances effectiveness by aligning incentives, sharing technical expertise, and facilitating problem-solving across the supply chain.

Monitoring and evaluation tools are essential for assessing whether fortification policies are achieving their intended nutritional outcomes. Modeling tools are commonly used to estimate population-level impacts and guide policy adjustments; however, these approaches rely on assumptions about consumption patterns and compliance that may not reflect real-world conditions. In this context, the premix reconciliation calculation represents an under-utilized but potentially powerful method for assessing compliance. By comparing the quantity of premix procured by producers against reported production volumes, this approach provides a more direct and objective estimate of both producer-specific and national compliance levels (6). Wider adoption of this method could substantially improve accountability and evidence-based decision-making.

Despite the availability of regulatory frameworks and monitoring tools, inadequate compliance by millers and other food processors remains a major constraint to policy effectiveness. High fortification costs particularly the recurring expense of premix, equipment maintenance, and quality assurance pose significant barriers, especially for small and medium-scale processors operating with narrow profit margins. Technical capacity gaps, including limited knowledge of fortification standards, insufficient laboratory facilities, and lack of trained personnel, further undermine consistent implementation. These challenges are often compounded by weak monitoring systems, infrequent inspections, and limited enforcement, which reduce incentives for compliance.

In addition, low consumer awareness of fortified foods diminishes market-driven demand for compliant products, reducing pressure on producers to adhere to standards. Supply-chain disruptions, including inconsistent premix availability and logistical challenges in rural or import-dependent settings, further compromise fortification quality and continuity. As a result, mandatory fortification policies may exist in law but fail to deliver meaningful nutritional impact in practice. Studies indicate that many local companies perceive fortification as technically complex and financially burdensome, leading to partial, inconsistent, or complete non-compliance (26).

Table 7 shows a policy comparison summary for Malawi and Mozambique.

**Table 7:** Summary of Malawi and Mozambique policy comparison.

Aspect	Malawi	Mozambique
Start of programming	Early 2000s (pilots) → 2011 standards	2010 planning → 2016 decree
Legal footing	Gazette mandates & regulatory standards	Council decree; detailed mandates
Coverage	Maize flour, wheat flour, oil, sugar, salt, plus MNPs	Maize flour, wheat flour, oil, sugar, salt
Policy clarity	Clear standards, enforcement protocols	Precise micronutrient specifications, timeline
Stakeholders	NFA, Ministries, NGOs, donors	CONFAM, ministries, NGOs, donors
Enforcement capacity	Bureau of Standards inspections, MOMT	Inspector training in progress, baseline data
Rural reach	MNP home-based fortification pilots and small hammer mills	Small-producer exemptions apply
Comprehensiveness	Broad but evolving reach to rural areas	Extensive, but needs full rollout and stronger enforcement mechanisms

#### 3.6.5 Best practices identification

Early adoption/adaptation of mandatory legislation with monitoring for Malawi in 2011 (38) for multiple staples, backed by district inspections, achieved high compliance and coverage. Mozambique’s (34) decree, on the other hand, came after years of implementing a voluntary fortification program. It is therefore our belief that early adoption or adaptation of interventions allows for learning and improvement of implementation nuances. Additionally, as much as the voluntary start pushed an extensive reach for Mozambique, it left gaps in monitoring and evaluation structures that only became worse with the introduction of mandatory fortification. Mandatory fortification does not require consumers to change food purchasing preferences, distributes the health benefits more equitably than voluntary fortification across a population, establishes safe levels of included nutrients, and is not subject to the food manufacturers’ marketing investments or discretion (36).

Mozambique’s economy is gaining momentum in a challenging global context. Economic recovery has gathered pace, with growth reaching 4.1% in 2022, despite the worsening global economy, and inflation hit a five-year high as global fuel and food prices surged, and adverse weather reduced domestic food production (39). Mozambique’s economic performance deteriorated markedly in 2024, annual real GDP growth declined to 2.2% in 2024 from 5.5% in 2023 primarily attributable to post-election protests and climate-related shocks, including Cyclone Chido and the El Niño phenomenon (40). Since 2020, Malawi’s economy has experienced a deep and protracted economic crisis, and economic growth rates have dropped from an average of 4.1% (2011–2019) to 2.2% since 2020. While external shocks like cyclones, droughts, and geopolitical instability have adversely affected Malawi, neighboring countries have tended to recover more quickly from the same shocks, but Malawi’s challenges have been compounded by long-standing policies that have contributed to widening fiscal and current account deficits. Inflation remains elevated, Malawi’s private sector is facing a challenging business environment, and the high cost of borrowing further limits investment and expansion opportunities (41). According to Resnick (42), political economy factors within and across countries intersect with LSFF efforts and sometimes undermine progress (Incoherent trade, tax, and macroeconomic policies in a context of multiple shocks and crises, protectionist impulses aimed at building up domestic agro-industries, lack of financial commitment to LSFF structures in the absence of donor support, and uneven contributions). In that context, the best policy is to introduce and push for LSFF in windows of a relatively stable economy, as this facilitates easier uptake by the food industry.

Providing exemptions for Small and Medium Enterprises, in the case of maize flour, fortification is not mandatory whenever it is produced by small-scale mills which provide milling services exclusively for family consumption (30) Small-scale exclusions cause coverage gaps (Mozambique); inadequate monitoring during crises (for example, COVID) disrupts programs. High costs and quality gaps require ongoing investment. Lessons emphasize policy support and consumer education. Complementarity with other strategies has also been shown to be an effective method in improving public health outcomes. Combining fortification with dietary diversification enhances nutritional outcomes, for example, orange-fleshed sweet potatoes and vitamin A supplementation (14, 16). Interventions must be carefully designed to complement other interventions covering the same problem; however, to prevent issues of redundancy and hypervitaminosis (29).

## 4 Discussion

### 4.1 Food processing and value addition

Value addition through food fortification in Malawi and Mozambique transforms low-value staple processing into nutrition-sensitive industrial activity by embedding micronutrients into widely consumed foods (oil, salt, sugar, maize and wheat flour), thereby raising both the public-health and market value of processed products and creating opportunities for millers and processors to add a certified “fortified” attribute to their brands. Evidence from Malawi’s national program shows fortified oil and sugar substantially improved vitamin A supply in modeled dietary scenarios and coincided with reductions in vitamin A deficiency, illustrating the nutritional benefit that underpins the economic value addition (35). In Mozambique, mandatory fortification standards for flours, oil, sugar and salt have expanded the role of commercial millers and packers in national nutrition strategies, but program evaluations also note uneven regulatory enforcement and coverage gaps that limit how fully value is captured across the value chain (33, 43).

### 4.2 Divergent policy approaches and influencing factors

Malawi and Mozambique have adopted different approaches to food fortification as a result of their unique governance structures, economic priorities, and donor dynamics. Malawi’s approach is characterized by centralized policy coordination, relatively strong institutional frameworks (for example, the Malawi Bureau of Standards), and consistent donor engagement (44). Conversely, Mozambique has leaned toward a more decentralized, multisectoral model, exemplified by CONFAM (the National Committee for Food Fortification), which fosters inclusive stakeholder involvement but often lacks consistent regulatory enforcement (14). Donor influence has played a substantial role in both contexts, yet in different ways (7). In both countries, donors such as GAIN and UNICEF have supported mandatory policy rollout, capacity building, and monitoring systems. Donor engagement has been broader but sometimes fragmented, contributing to variations in implementation across regions (45). These findings highlight the role of institutional governance and policy coherence in shaping national fortification strategies.

### 4.3 Effectiveness of mandatory vs. voluntary approaches

Mandatory fortification policies tend to yield higher levels of compliance and wider population coverage than voluntary initiatives (5, 36). Malawi’s legal requirements for fortification of sugar, oil, and flour have led to consistent product availability in the market, particularly in urban areas. By contrast, Mozambique initially adopted voluntary guidelines, which resulted in limited adoption among producers until more stringent enforcement mechanisms were introduced. Mandatory policies offer legal clarity and compel compliance, especially when supported by monitoring systems and penalties. Voluntary schemes, while more flexible, often fail to motivate producers, especially in the absence of consumer demand or economic incentives (46).

### 4.4 Role of regulatory enforcement

A strong correlation exists between regulatory enforcement mechanisms and compliance rates (5). Malawi’s policy environment includes structured inspection protocols, penalties for non-compliance, and periodic product testing. Mozambique, while making progress with digital monitoring tools like FortifyMIS, still faces challenges in terms of logistical reach and institutional capacity, particularly at the provincial level. Compliance rates are highest among large-scale producers who have the resources to meet regulatory standards (19). However, limited enforcement in informal markets and rural areas undermines national coverage goals for both countries (47). These findings underscore the need for well-resourced and decentralized enforcement strategies to improve overall fortification effectiveness.

### 4.5 Economic incentives and industry engagement

The presence of economic incentives, such as subsidies for premix purchases and tax exemptions on equipment, significantly affects industry compliance and investment (11). In Malawi, such incentives have facilitated technology uptake and operational adjustments among food processors. Mozambique has seen less consistent application of economic incentives, which partially explains the slower response from small and medium enterprises (SMEs). Reducing financial and regulatory barriers that constrain fortification by the food industry, such as excessive registration, taxes on fortification inputs, and onerous reporting requirements (48). Where supportive financial environments exist, producers are more likely to perceive fortification not as a regulatory burden but as a feasible business investment. This highlights the importance of aligning fortification goals with broader economic development strategies.

### 4.6 Public-private partnerships and policy sustainability

Public-private partnerships (PPPs) have proven instrumental in enhancing the sustainability and effectiveness of fortification programs (11). Partnerships with NGOs and private processors have strengthened policy implementation through co-financing, capacity building, and technical assistance (49). Mozambique’s CONFAM structure serves as a promising PPP model, though its impact has been uneven due to limited accountability mechanisms. Successful PPPs share common features: mutual trust, clear roles, and long-term funding commitments. When effectively managed, they offer a platform for dialog, innovation, and joint problem-solving, enhancing resilience and adaptability.

### 4.7 SME capacity building and compliance

Evidence shows that fortification policies, inclusive of SME capacity building, lead to higher compliance rates. Malawi has initiated pilot projects to provide technical training and low-cost equipment to SMEs, resulting in improved adoption rates (26). Mozambique has experienced slower progress in this area, although recent donor-funded initiatives have begun targeting SMEs in rural regions. Without specific support, SMEs often lack the resources, knowledge, and motivation to implement fortification, creating significant coverage gaps (50). Inclusive policy design that addresses SME needs is essential for equitable public health outcomes.

### 4.8 Policy timing and economic stability

The timing of policy introduction relative to macroeconomic conditions significantly influences implementation success (17). Political economy factors within and across countries intersect with LSFF efforts and sometimes undermine progress, for example, incoherent trade, tax, and macroeconomic policies in a context of multiple shocks and crises, protectionist impulses aimed at building up domestic agro-industries, lack of financial commitment to LSFF structures in the absence of donor support, and uneven contributions (42). In periods of economic stability, governments and industries alike are better positioned to absorb the costs of compliance and invest in infrastructure. Malawi’s fortification rollout faced delays during times of economic uncertainty, while Mozambique made key policy strides during relatively stable periods. This finding emphasizes the value of timing policy reforms to coincide with fiscal and institutional readiness, thereby enhancing uptake and sustainability (17).

### 4.9 Institutional capacity and policy effectiveness

Despite similar socioeconomic profiles, Malawi and Mozambique display marked differences in policy effectiveness due to variations in institutional capacity. Malawi’s clearer legislative framework, dedicated fortification units, and relatively stable governance have enabled more consistent policy application; however, not fully. Policy incoherence is one of the critical issues affecting the potency of most policies on inequality in Malawi (51). There are several policies whose sectoral objectives and goals do not complement the goals in other sectors, thereby eroding their impact. Furthermore, a lack of coordination between institutions in the formulation and implementation of interrelated policies has also led to policy incoherence and weak inequality outcomes (52). Comparative evidence suggests that public service provision in rural areas can be improved by decentralization; in fact, it is not clear that there are ready reforms other than decentralization that will provide public services on the massive scale that Mozambique’s rural areas seem to be on the point of demanding (53) Mozambique’s challenges with decentralization, bureaucratic turnover, and resource limitations have impeded policy cohesion and operational follow-through. These findings highlight institutional strength as a critical determinant of fortification success.

### 4.10 Regional trade harmonization and market access

Review the relevance and appropriateness of fortification standards and program design (in light of national changes and regional or global recommendations) and make adjustments as necessary (17). Mozambique’s alignment with regional trade standards through SADC has improved cross-border food trade and market access for fortified products. Harmonization reduces regulatory barriers and enhances economies of scale, encouraging producers to invest in fortification. Malawi, although internally well-regulated, has not prioritized regional integration to the same extent, potentially limiting export potential (54). Increased harmonization can incentivize compliance while fostering broader public health gains across the region.

The comparative analysis confirms several key hypotheses, demonstrating that effective fortification policies require a combination of strong governance, mandatory legislation, strategic incentives, and inclusive implementation approaches. Differences in institutional capacity, donor engagement, and economic contexts have shaped distinct fortification trajectories in Malawi and Mozambique. These insights underscore the need for context-specific strategies that align technical interventions with political, economic, and social realities.

## 5 Recommendations

Based on the findings and conclusions of this study, the following recommendations are proposed for policymakers, development partners, and industry stakeholders:

In Malawi, mandatory fortification is primarily operationalized through food standards enforced by the Malawi Bureau of Standards in collaboration with the Ministry of Health and the National Fortification Alliance. While this framework is well established, its effectiveness is constrained by limited inspection capacity, fragmented monitoring across agencies, and weak enforcement at both production and import levels. Addressing these challenges requires a shift toward risk-based regulatory approaches, greater use of cost-effective compliance tools such as premix reconciliation, and strengthened coordination with industry associations and civil society actors to extend surveillance beyond formal inspections. Furthermore, the high costs and technical demands of fortification for small and medium-scale millers present a significant barrier to compliance; these challenges could be mitigated through targeted technical assistance, shared fortification infrastructure, and temporary financial incentives to offset premix and equipment costs.

In Mozambique, fortification is governed through a centralized legal framework under Decree No. 9/2016 and coordinated by the National Food Fortification Program (CONFAM), providing strong policy coherence. However, implementation is challenged by uneven compliance among producers, limited quality assurance capacity, and weak monitoring of fortified foods at border points and in informal markets. Strengthening inter-institutional coordination between CONFAM, customs authorities, and food inspectors, expanding routine market sampling, and improving data systems for compliance tracking are therefore critical. For smaller processors, particularly in rural and peri-urban areas, phased enforcement combined with targeted training and integration of fortification support into existing nutrition and industrial development programs may reduce resistance and improve uptake.

Challenges in implementation of these recommendations can be mitigated by involvement of all stakeholders from planning stage, emphasizing transparency, commitment and shared ownership of public health interventions.

## 6 Conclusion

This systematic review came to the following key conclusions. Malawi and Mozambique have adopted differing fortification strategies, shaped by their respective governance structures, donor relationships, and economic priorities. Differences in institutional coherence and administrative efficiency between Malawi and Mozambique account for variations in policy outcomes. Generally, legal mandates for fortification are more effective than voluntary approaches in achieving widespread compliance and coverage. Gaps in enforcement capacity in both countries undermine national coverage goals. Subsidies, tax exemptions, and donor support also influence processors’ willingness and ability to invest in fortification. Collaborative governance models, public-private partnerships, and inclusive policies (for example, supporting small and medium enterprises) enhance sustainability, promote transparency, and ensure that private sector capabilities are effectively leveraged. Fortification policies introduced during periods of economic growth or stability are more likely to succeed due to increased fiscal space, institutional capacity, and reduced resistance from industry. In conclusion, the effectiveness of food fortification policies is determined not only by technical considerations but by the interplay of governance, economics, stakeholder dynamics, policy timing, donor requirements and how the countries respond to these aid stipulations.

## Data Availability

The original contributions presented in the study are included in the article/Supplementary material, further inquiries can be directed to the corresponding author.
